# 25, 50 and 75 years ago

**DOI:** 10.1111/ans.70094

**Published:** 2025-03-14

**Authors:** Julian A. Smith

**Affiliations:** ^1^ Department of Surgery Monash University Melbourne Victoria Australia

## 25 years ago

### McDermott FT, Cordner SM, Tremayne A, and the consultative committee on road traffic fatalities in Victoria. *ANZ J Surg.* 2000;70:710–721

Since 1992, the Consultative Committee on Road Traffic Fatalities in Victoria has identified deficiencies and errors in the management of 559 road traffic fatalities in which the patients were alive on arrival of ambulance services. The Committee also assessed the preventability of deaths. The reproducibility of results using its methodology has been shown to be statistically significant.

The Committee's findings and recommendations, the latter made in association with the learned Colleges and specialist Societies, led to the establishment of a Ministerial Taskforce on Trauma and Emergency Services. As a consequence, in 2000, a new trauma care system will be implemented in Victoria. This paper presents a case example demonstrating the Committee's methodology. The Committee has two 12‐member multidisciplinary evaluative panels. A retrospective evaluation was made of the complete ambulance, hospital, and autopsy records of eligible fatalities. The clinical and pathological findings were analysed using a comprehensive data proforma, a narrative summary, and the complete records. Resulting multidisciplinary discussion problems were identified, and the potential preventability of death was assessed.

In the present case example, the Committee identified 16 management deficiencies, of which 11 were assessed as having contributed to the patient's death; the death, however, was judged to be non‐preventable. The presentation of this example demonstrating the Committee's methodology may be of assistance to hospital medical staff undertaking their own major trauma audit.

### Chambers AJ, Lord RSA. Management of gunshot wounds at a Sydney teaching hospital. *ANZ J Surg.* 2000;70:209–215

Injuries caused by firearms account for only a small percentage of trauma admissions and deaths in Australia but are frequently the subject of media and public attention. The present study examines the epidemiology and management of firearm injuries presenting to St Vincent's Hospital, which is located at the edge of the central business district of Sydney. The medical records of all patients presenting to St Vincent's with a gunshot wound (GSW) from January 1988 to December 1998 were analyzed. Additional details were acquired from New South Wales State Coroner's Court reports and media archives, especially major newspapers. Seventy‐four patients presented to St Vincent's Hospital with 103 GSWs. The age (mean ± SD) was 31 ± 11 years. Sixty‐seven patients (91%) were male. Alcohol was identified as a factor in 24 cases (32%) while other drugs were indicated in four cases (5%). Ten patients (14%) had intentionally self‐inflicted wounds, seven (9%) had accidental wounds, and 57 (77%) had wounds that were caused by crime‐related violence. Sixty patients (81%) underwent surgery for their injury. Thirty complications were seen in 18 patients (24%). Eleven patients (15%) died. The length of hospital stay (mean ± SEM) was 18 ± 9 days. The incidence of trauma due to firearms has not increased at St Vincent's Hospital in the period 1988–1998. Most GSWs were inflicted in the setting of criminal violence, with a high proportion due to handguns. Patients were mostly young men, and alcohol or other drugs were frequently involved. Outcomes are comparable to other centers managing large volumes of penetrating trauma.

## 50 years ago

### Lee WKC, Morris PJ. Arterial embolectomy in the lower limbs. *ANZ J Surg.* 1975;45:136–139

A series of arterial embolectomies of the lower limb is reviewed, and the results are compared with those in a previous series reported from this hospital. There were 40 emboli in the 36 patients, with an operative mortality of 16% and a limb salvage rate of 85%. This represents a considerable improvement on the results reported from the first series and is attributed to the greater experience of surgeons with the Fogarty embolectomy catheter. However, there has been no decrease in the delay between the onset of symptoms and the embolectomy. If this aspect of management could be improved, then even better results should be obtained. Doctors, both within and outside hospitals, must be made more aware of the need for urgency in the management of arterial emboli of the lower limbs, for non‐operative treatment with anticoagulants has no place in the management of this condition.

### King B, McIntyre R, Myers K. Below‐knee amputation in peripheral arterial disease. *ANZ J Surg*. 1975;45:301–304

**Fig. 1:**
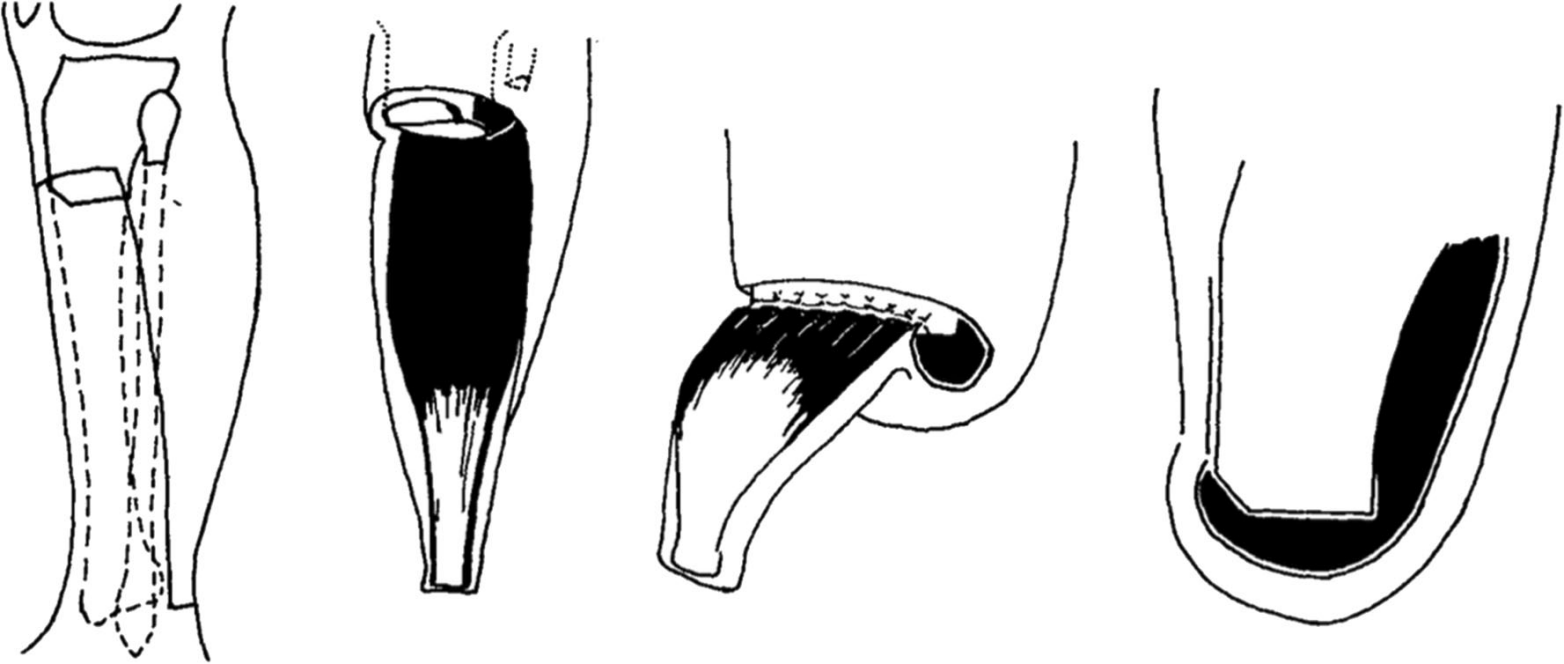
(Left) skin incision and level of bone section; (Left Centre) posterior flap including gastrocnemius and soleus; (Right Centre) fascia of posterior flap sutured to periosteum and anterior tibial fascia; (Right) completed amputation.

A below‐knee amputation will heal in most patients with atherosclerotic peripheral arterial disease, using no anterior flap and a long posterior myoplastic flap. The technique used by the authors (Fig. 1) is presented in detail, with their experience in 32 cases. Satisfactory healing can be achieved with a below‐knee amputation in most severely ischaemic limbs. The elimination of an anterior flap with the construction of a long myoplastic posterior flap and the use of a non‐traumatic technique allow most below‐knee amputations to heal. The healed stump is well‐padded and suitable for the fitting of a prosthesis. If dead muscle is present at the level of amputation, an above‐knee amputation should be performed rather than an attempt to excise the muscles at a more proximal level.

## 75 years ago

### King T. The treatment of traumatic ulnar neuritis. Mobilization of the ulnar nerve at the elbow by removal of the medial epicondyle and adjacent bone. *ANZ J Surg.* 1950;20:33–42

In summary:

1. The medial epicondyle and adjacent bone (diaphysis) were deliberately excised in 16 patients with ulnar neuritis and for other causes including irritation in the groove, cubitus valgus and varus, as a preliminary step for arthroplasty of the elbow, for repair of ruptured triceps; for ununited fracture of the olecranon and also when the ulnar nerve had to be sutured at the wrist and there was a three‐inch gap to be bridged. Skiagrams of five illustrative cases are shown.
2. The results have been satisfactory. The minor neurological changes cleared up, and where there were gross signs, there was either improvement or no further progress of nerve degeneration, and pain disappeared.
3. This simple operation appears to avoid the danger of fibro‐cicatrical contracture, which occasionally occurs when the nerve is embedded in the flexor muscles and, perhaps, minimizes the danger of pressure upon it by external objects as can take place if the nerve is transposed anteriorly and subcutaneously.

